# Identification of Common Brain Protein and Genetic Loci Between Parkinson's Disease and Lewy Body Dementia

**DOI:** 10.1111/cns.70370

**Published:** 2025-04-09

**Authors:** Tingting Jia, Fuhua Yang, Fengqin Qin, Yongji He, Feng Han, Chengcheng Zhang

**Affiliations:** ^1^ Mental Health Center and Psychiatric Laboratory, the State Key Laboratory of Biotherapy West China Hospital of Sichuan University Chengdu Sichuan China; ^2^ Department of Gastroenterology and Hepatology and Sichuan University‐University of Oxford Huaxi Joint Centre for Gastrointestinal Cancer West China Hospital of Sichuan University Chengdu Sichuan China; ^3^ Department of Nephrology The Sixth People's Hospital of Chengdu Chengdu Sichuan China; ^4^ Department of Neurology The 3rd Affiliated Hospital of Chengdu Medical College Chengdu Sichuan China; ^5^ Clinical Trial Center, National Medical Products Administration key Laboratory for Clinical Research and Evaluation of Innovative Drugs West China Hospital Sichuan University Chengdu China; ^6^ Department of Emergency Medicine Hainan General Hospital, Hainan Affiliated Hospital of Hainan Medical University Haikou China

**Keywords:** dementia, protein quantitative trait loci, proteome‐wide association study, risk gene

## Abstract

**Background:**

Parkinson's disease (PD) and Lewy body dementia (LBD) have many common features, including clinical manifestations, neurochemistry, and pathology, but little is known about their shared brain proteins and genetic factors.

**Methods:**

To identify susceptibility‐related brain proteins that are shared between PD and LBD patients, proteome‐wide association studies (PWASs) were conducted by integrating human brain protein quantitative trait loci (pQTLs) with large‐scale genome‐wide association studies (GWASs) of both diseases. Subsequently, pleiotropy‐informed conditional false discovery rate (pleioFDR) analysis was performed to identify common risk genetic loci between PD and LBD. Finally, the downregulation of these risk genes in different disease states was validated by differential gene expression analysis.

**Results:**

PWASs identified 12 PD risk proteins and nine LBD risk proteins, among which *TMEM175* (*z*_PD_ = −7.25, *P*_PD_ = 4.12E‐13; *z*_LBD_ = −6.02, *P*_LBD_ = 1.75E‐09) and *DOC2A* (*z*_PD_ = −4.13, *P*_PD_ = 3.71E‐05; *z*_LBD_ = −3.91, *P*_LBD_ = 9.08E‐05) were shared. PleioFDR analysis revealed that five genetic risk loci mapped to eight genes associated with PD and LBD, including the proteome‐wide significant risk gene *TMEM175* (ConjFDR = 5.74E‐03). Differential expression analysis verified that *TMEM175* was significantly downregulated in the midbrains of PD patients (*p* = 1.19E‐02), and further exploration revealed that *TMEM175* was also dramatically downregulated in the substantia nigra of PD patients (*p* = 1.16E‐02) and incidental Lewy body disease patients (*p* = 7.52E‐03). Moreover, *TMEM175* was significantly downregulated in induced pluripotent stem cell‐derived dopaminergic neurons from PD patients (*p* = 4.60E‐02).

**Conclusion:**

Dysregulation of *TMEM175* may confer PD and LBD risk and may be partly responsible for their comorbidity. Our results revealed the common genetic risk factors between PD and LBD, which elucidated the shared genetic basis of these diseases.

## Graphical abstract

Dysregulation of *TMEM175* may confer Parkinson's disease and Lewy body dementia risk and partly cause comorbidity.

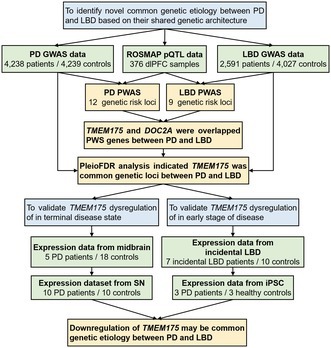

## 1 Introduction

Parkinson's disease (PD) is the second most common neurodegenerative disease affecting more than 6 million people worldwide [1]; Lewy body dementia (LBD) is the second most common neurodegenerative dementia, accounting for approximately 5% of all dementia patients among older populations [2]. These two diseases share common features in terms of clinical manifestations, neurochemistry, and neuropathology, including progressive cognitive impairment, parkinsonism, and Lewy body deposition, which makes it difficult to distinguish PD from LBD [3]. Therefore, PD and LBD are considered to be on the same disease spectrum, with the main difference being the timing of cognitive impairment onset (symptoms of dementia usually precede parkinsonism in LBD patients, whereas the onset of cognitive impairment often occurs after the onset of motor symptoms in PD patients) [4]. Genetic factors are key in the pathogenesis of PD and LBD, with estimated single‐nucleotide polymorphism (SNP) heritabilities of 22% [5] and 59.9% [6], respectively, highlighting the pattern of polygenic inheritance. Through genome‐wide association studies (GWASs), several risk loci for PD [5, 7] and LBD [8, 9] have been identified, illustrating the association between pathogenic SNPs and disease phenotypes, but how these loci confer disease risk through multilevel regulation remains unclear. Brain proteins, such as α‐synuclein and amyloid‐β, and the potential synergistic relationship between them play important roles in the pathogenesis of PD and LBD [10, 11]. However, antibody therapies targeting these proteins have performed poorly in clinical trials [12, 13], indicating the necessity of identifying other novel brain proteins as therapeutic targets. Proteome‐wide association studies (PWASs) use protein quantitative trait loci (pQTLs) to measure the effects of risk genes on disease phenotypes at the protein level [14]. Proteins derived from the dorsolateral prefrontal cortex (DLPFC) have been selected for these studies because the DLPFC has been strongly associated with impaired cognition [15], a key symptom considered in the diagnosis of these two diseases. Moreover, substantial transcriptional and translational alterations in PD and LBD patients have been demonstrated in this brain region [16, 17, 18].

Pairwise linkage disequilibrium score regression (LDSC) performed in a previous study revealed that PD and LBD had a positive genome‐wide genetic correlation (rg = 0.6352) [19], providing the first evidence for a global genetic correlation between PD and LBD. Pleiotropy‐informed conditional false discovery rate (pleioFDR) analysis, a multitrait analysis based on the empirical Bayesian statistical framework that improves the statistical power of single‐trait GWASs, has been applied to the identification of shared risk loci among different diseases [20]. The aim of the current study was to use the pleioFDR approach with LBD and PD GWASs to identify shared pleiotropic loci as intervention targets to prevent or treat these diseases simultaneously.

A common feature that is specific to PD and LBD is the neurodegeneration of dopaminergic (DA) neurons in the substantia nigra in the midbrain [21]. Therefore, the substantia nigra of the postmortem brain is an ideal tissue source, as this region can directly reflect gene expression alterations in disease‐affected brain regions. To explore whether the dysregulation of risk genes occurs in the early stages of the disease, we examined the expression of the risk genes in the substantia nigra of patients with incidental Lewy body disease. Previous stereologic and nonstereologic investigations have provided evidence that incidental Lewy body disease is the preclinical phase of PD/LBD and exhibits few clinically relevant motor and cognitive symptoms [22, 23, 24]. Thus, incidental Lewy body disease has been suggested to represent the initial state in the Lewy body disease spectrum before the development of the more severe PD and LBD [3]. Additionally, we investigated the expression of risk genes in differentiated induced pluripotent stem cell‐derived neurons. Unlike postmortem brain tissue, induced pluripotent stem cells (iPSCs) obtained by reprogramming patient‐derived cells can differentiate into dopamine neurons. After the removal of epigenetic alterations by reprogramming, iPSC‐derived neurons retain the patient's genetic composition and can represent the earliest stages of the disease process [25, 26]. Combining the strengths of different disease stages and tissues from different sources, we aimed to verify the downregulation of risk genes in the preclinical and clinical states.

**FIGURE 1:**
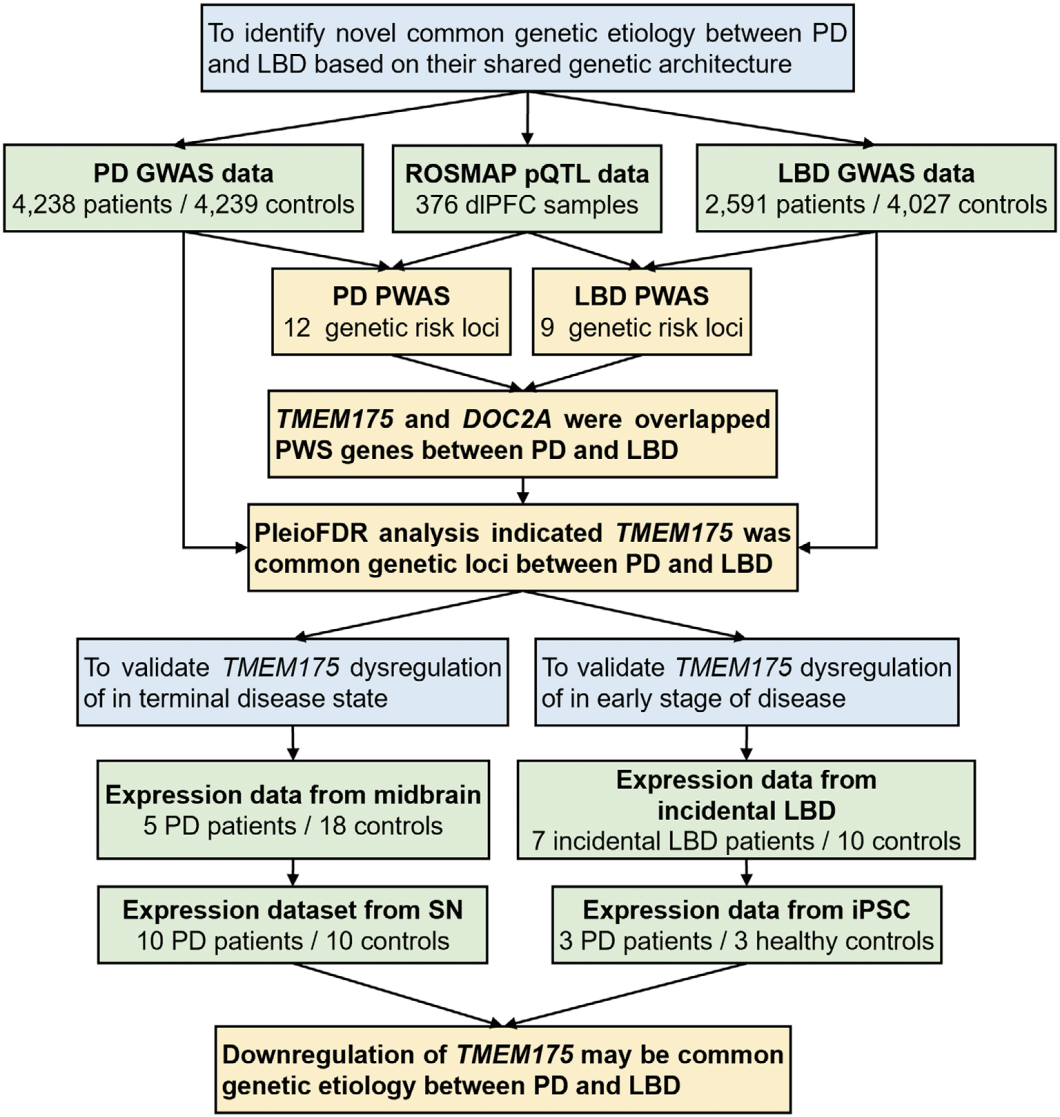
Schematic workflow of the present study. The schematic framework used in the present study revealed the common risk genes of PD and LBD at multiple levels. First, a PWAS was performed using pQTL data (ROSMAP dataset) and summary statistics from large‐scale GWASs of the two diseases to discover common susceptibility proteins. We subsequently performed pleioFDR analysis and identified loci associated with both PD and LBD at the SNP level. Finally, differential expression analysis of the human brain and iPSC‐derived tissues validated the downregulation of *TMEM175* in different disease states compared with healthy controls. GWAS, genome‐wide association study; iPSC, induced pluripotent stem cell; SNP, single‐nucleotide polymorphism; LBD, Lewy body dementia; PD, Parkinson's disease; pleioFDR, pleiotropy‐informed conditional false discovery rate; pQTL, protein quantitative trait locus; PWAS, proteome‐wide association study; ROSMAP, Religious Orders Study and Rush Memory and Aging Project; SN, substantia nigra.

The overall analysis workflow applied in this study is summarized in Figure 1. In brief, we first conducted PWASs using large‐scale GWAS summary statistics of PD and LBD and the ROSMAP pQTL dataset to identify PD and LBD risk genes. Next, we applied the pleioFDR method to the PD and LBD GWAS summary statistics to identify risk loci shared between both diseases. Third, differential expression analysis was performed using the substantia nigra from postmortem brains and iPSC‐derived differentiated neurons from patients and healthy controls to validate the dysregulation of these shared risk genes at the transcriptional level. In summary, we identified novel PD and LBD risk proteins, as well as shared loci that play important roles in the genetic overlap of the two diseases, thus providing new insights into their pathogenesis, which may aid in the development of new prevention or treatment options.

## 2 Methods

### 2.1 GWAS for Parkinson's Disease

The PD GWAS summary statistics used in the present study consisted of data from 4238 PD patients and 4239 controls, including GWAS data obtained from dbGaP and two datasets obtained directly from investigators. All PD patients were diagnosed according to standard UK Brain Bank criteria [27], allowing the inclusion of patients with a family history of PD. After genotyping using the Illumina array, data cleaning, quality control and imputation, and logistic regression adjusted for the confounding factors of sex and age were performed. Principal component analysis was used to obtain principal components, which were also included in the regression models to adjust for ancestry differences (i.e., to mitigate the risk of population stratification). An inverse‐variance weighting scheme was subsequently applied in the meta‐analysis using METAL (http://www.sph.umich.edu/csg/abecasis/Metal/). More details on sample selection, quality control, and data analysis can be found in the original article [28].

### 2.2 GWAS for Lewy Body Dementia

The GWAS summary statistics of Lewy body dementia were obtained from a recently published large‐scale GWAS meta‐analysis that included 2591 LBD patients and 4027 neurologically healthy aged controls from three cohorts of European descent. All LBD patients were diagnosed based on the presence of pathologically confirmed or clinically probable disease according to consensus criteria [29, 30]. After each cohort was subjected to quality control procedures, genotypic data were analyzed with logistic regression in PLINK [31]. A detailed description of the samples and variant quality control measures has been published previously [9].

### 2.3 pQTL Data

Human brain protein quantitative trait loci data were obtained from the Religious Orders Study and Rush Memory and Aging Project (ROSMAP) [32], a dataset collected through the analysis of dorsolateral prefrontal cortex tissue in 400 individuals of European ancestry, of which 40.7% of participants had no cognitive impairment. After the batch effects were removed, isobaric tandem mass tag (TMT) peptide labeling was used for proteomic sequencing, and liquid chromatography coupled with mass spectrometry (LC–MS) was used for peptide analysis. Genotyping was accomplished using either the Illumina OmniQuad Express or Affymetrix GeneChip 6.0 platform. Normalized abundances were calculated for 8356 proteins from 376 individuals, of which 1475 proteins showed significant cis associations with genetic variation. These 1475 protein weights were used for the subsequent PWASs. The detailed quality control process was described in the primary study [33].

### 2.4 PWASs

PWASs were performed using FUSION (http://gusevlab.org/projects/fusion) [14]. Briefly, protein reference weights for the ROSMAP dataset were estimated using multiple prediction models (top1, blup, lasso, enet, bslmm) to calculate the effects of SNPs on protein abundance. The SNP‐based genetic effects on the disease (summary statistics of PD or LBD GWAS) and the protein weights were subsequently combined by calculating the linear sum of the z score × weight for the independent SNPs at each locus.

### 2.5 PleioFDR Analysis

To identify shared susceptibility loci between PD and LBD, we performed pleiotropy‐informed conditional false discovery rate (pleioFDR) analysis [34], including conditional FDR (condFDR) and conjunctional FDR (conjFDR) analyses. Briefly, conditional quantile–quantile (Q–Q) plots were constructed to assess the cross‐disease enrichment of polygenic effects. CondFDR was performed using the associations between genetic variants and the conditional trait (trait 2) to rerank SNPs and recalculate the associations between these variants and the primary trait (trait 1). The conjFDR statistic was defined as the maximum of the paired condFDR values of SNPs that had *p* values lower than the observed *p* value for both the primary and conditional traits; this value was a conservative estimate for the posterior probability of the SNP not being associated with either trait. The significance threshold for conjFDR was set at 0.01.

### 2.6 Differential Expression Analysis

#### 2.6.1 Differential Expression Analysis in the Brain

We next performed differential expression analysis to verify the downregulation of the risk gene *TMEM175* at the transcriptional level in patients compared with healthy controls. First, we investigated dopaminergic neurons from the midbrain, followed by analysis of the substantia nigra to confirm the region where *TMEM175* is dysregulated. Normalized gene expression profiles from fresh snap‐frozen human postmortem tissue were obtained from three independent studies. (1) The midbrain tissues of 18 healthy controls and five PD patients from the Netherlands Brain Bank (NBB) were collected [35]. DA neurons were selected through a laser capture procedure on the basis of their location and the presence of neuromelanin. RNA‐seq libraries were prepared using SMART‐seq2 combined with Tn5 tagmentation, and the samples were sequenced on the Illumina HiSeq2000, HiSeq2500, or NovaSeq platform. The reads per kilobase gene model and million mapped reads (RPKM) were generated by rpkmforgenes. Differential gene expression analysis was performed using the R limma package. (2) The substantia nigra tissues of 10 PD patients and 10 controls were obtained from the NBB [36]. After total RNA extraction and quality control, each substantia nigra sample was pooled from two individuals. All the samples were subsequently sequenced via HiSeq v4. FASTQC and Salmon were used for quality control and comparison after sequencing. Differential expression analysis was performed with the R DEseq2 package. (3) The RNA‐seq dataset containing the gene expression data of 12 PD patients, seven incidental Lewy body disease patients, and 10 controls without any neurodegenerative disease was provided by the Banner Sun Health Research Institute [37]. Neuromelanin‐positive neurons (DA neurons) from the substantia nigra were identified in sections of the ventral midbrain using the Leica LMD7000 system. After RNA extraction and quality control, total RNA sequencing was performed on an Illumina HiSeq 2000 platform. RPKMs were calculated for each transcript using SAMtools and rpkmforgenes. Differential expression of *TMEM175* was identified using one‐way ANOVA after confirmation of the normality of the data distribution using the Kolmogorov–Smirnov test. A *p* value of < 0.05 was used to determine whether there was a significant difference in expression between patients and controls in all datasets.

#### 2.6.2 Differential Expression Analysis in iPSCs

Human skin fibroblasts were obtained from three PD patients with *PARK2* mutations and three healthy donors [38], cultured as iPSCs, and induced to differentiate into neural progenitor cells (identified by Sox1 antibody staining) and terminally differentiated neurons (identified by beta‐III‐tubulin and tyrosine hydroxylase antibody staining). After total RNA extraction and quality control, the samples were sequenced using the Illumina NextSeq 500 System, and Trimmomatic and Salmon were subsequently used to trim the RNA‐seq data and quantify the reads. Differential expression analysis was performed using the R limma package. More information on the sample collection of expression datasets, generation of expression profiles, and data analysis can be found in previous publications [35, 36, 37, 38].

## 3 Results

### 3.1 PWASs Identified 12 Proteome‐Wide Significant Risk Genes for Parkinson's Disease and Nine Genes for Lewy Body Dementia, Among Which TMEM175 and DOC2A Overlapped

A total of 12 proteome‐wide significant (PWS) PD susceptibility genes were identified by PWAS by integrating the ROSMAP proteome reference weights with PD GWAS summary statistics. The cis‐regulated protein levels of these genes in the brain were significantly associated with PD risk. The upregulation of *TMEM163*, *STX4*, *RAB7L1*, and *ITGAM* (positive PWAS z score) and the downregulation of *TMEM175*, *DOC2A*, *CTSB*, *ASPHD1*, *CD38*, *DGKQ*, *AKR1C1*, and *PARK2* (negative PWAS z score) were associated with increased PD risk. Using the same method, nine candidate genes for LBD were identified in another PWAS, among which the upregulation of *WDFY1*, *STARD5*, and *TECPR2* and the downregulation of *TMEM175*, *DOC2A*, *NSF*, *RIPK2*, *TMED5*, and *EFNA3* were associated with increased LBD risk. Table 1 summarizes the detailed results of the PWAS analysis. Interestingly, we found that *TMEM175* and *DOC2A* were identified in the PWASs of both diseases, suggesting that these two genes may be involved in the common etiology of PD and LBD.

**TABLE 1:** Risk genes for Parkinson's disease and Lewy body dementia identified via proteome‐wide association study.

	Gene	CHR	Parkinson's disease		Lewy body dementia		Evidence of overlap
			PWAS z‐score	PWAS *p*	PWAS z‐score	PWAS *p*	
1	*TMEM175*	4	−7.25	4.12E‐13	−6.02	1.75E‐09	Yes
2	*DOC2A*	16	−4.13	3.71E‐05	−3.91	9.08E‐05	Yes
3	*TMEM163*	2	4.43	9.31E‐06			No
4	*CTSB*	8	−4.30	1.69E‐05			No
5	*ASPHD1*	16	−4.10	4.09E‐05			No
6	*CD38*	4	−4.10	4.12E‐05			No
7	*STX4*	16	4.09	4.22E‐05			No
8	*DGKQ*	4	−3.99	6.71E‐05			No
9	*AKR1C1*	10	−3.91	9.40E‐05			No
10	*RAB7L1*	1	3.64	2.70E‐04			No
11	*PARK2*	6	−3.59	3.32E‐04			No
12	*ITGAM*	16	3.58	3.42E‐04			No
13	*NSF*	17			−3.36	7.91E‐04	No
14	*WDFY1*	2			3.32	9.04E‐04	No
15	*STARD5*	15			3.26	1.11E‐03	No
16	*TECPR2*	14			3.19	1.45E‐03	No
17	*RIPK2*	8			−3.15	1.66E‐03	No
18	*TMED5*	1			−3.04	2.36E‐03	No
19	*EFNA3*	1			−3.03	2.44E‐03	No

*Note:* The table provides the z scores and their corresponding *p* values for proteome‐wide significant genes in the ROSMAP PWAS. A total of 12 significant genetic associations were identified for Parkinson's disease, and nine were identified for Lewy body dementia. *TMEM175* and *DOC2A* are shared between Parkinson's disease and Lewy body dementia. CHR, chromosome; PWAS, proteome‐wide association study.

Abbreviations: CHR, chromosome; PWAS, proteome‐wide association study.

### 3.2 PleioFDR Analysis Highlighted TMEM175 as a Common Genetic Risk Factor Between PD Patients and LBD Patients

**FIGURE 2:**
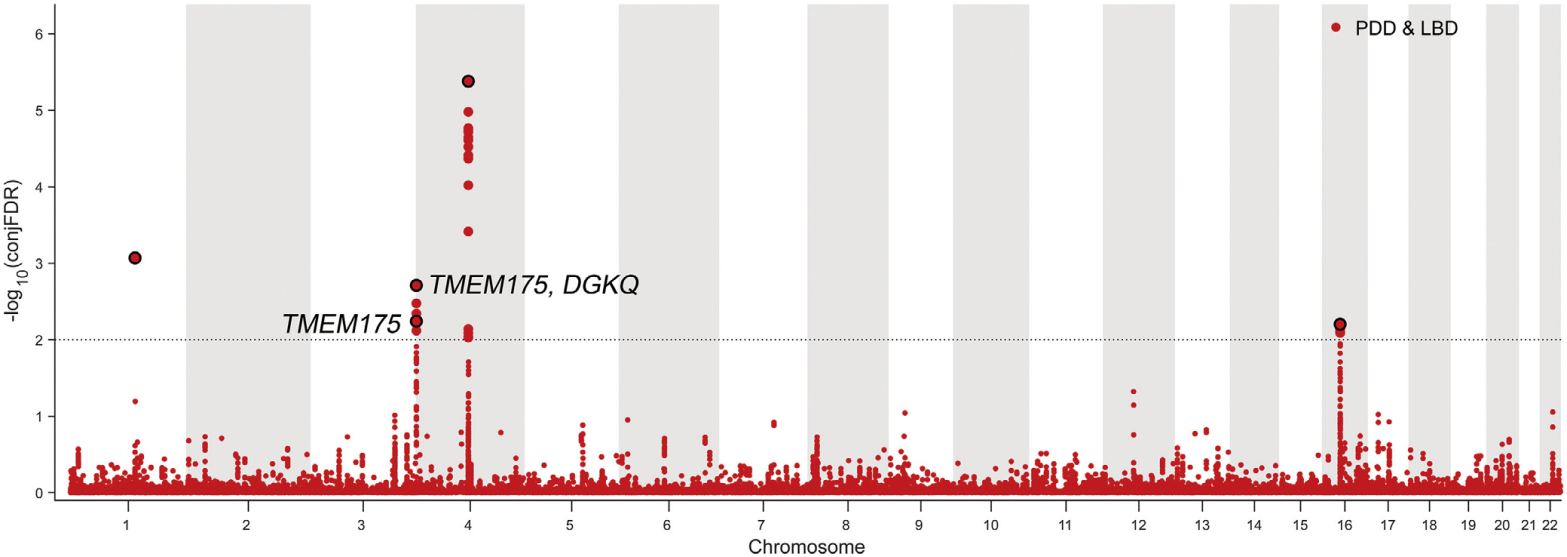
Manhattan plot showing common genetic variants jointly associated with PD and LBD at conjFDR < 0.01. Manhattan plot depicting the ‐log10‐transformed conjFDR values for each SNP jointly associated with PD and LBD on the y‐axis and the chromosomal location on the x‐axis. The dotted horizontal line represents the threshold for a significant association at conjFDR < 0.01. All independent lead SNPs are circled in black. Further details for these shared loci are available in Table S1. conjFDR, conjunctional false discovery rate; LBD, Lewy body dementia; PD, Parkinson's disease; SNP, single‐nucleotide polymorphism.

We used the pleioFDR approach to identify common genetic loci associated with PD and LBD. A conditional Q–Q plot revealed the presence of polygenic enrichment for PD in the given LBD strata (Figure S1A), manifested by a leftward shift from the expected baseline, indicating the potential common genetic basis between these two diseases. Stronger enrichment (a more rapid leftward shift) was observed as the significance of the association with the conditional trait increased; that is, more obvious deviations in SNP–PD associations from the empirical distribution occurred for the SNPs with stronger associations with LBD, supporting the hypothesis of a common genetic architecture. Similar results were observed with PD as the conditional trait and LBD as the primary trait (Figure S1B). To provide a map of the loci shared between PD and LBD, we performed pleioFDR analysis and identified a total of 34 SNPs (conjFDR < 0.01) at five unique loci (Table S1). These results were visualized in a Manhattan plot (Figure 2). From each of these loci, single SNPs with the minimum conjFDR value (signal of the strongest association) were selected as lead SNPs to represent their loci. All of the lead SNPs (rs6599389, rs1051613, rs2230288, rs1372519, and rs11150577 in Table 2) had a concordant direction of effect (as indicated by z score symbols) for PD and LBD, which implies that these genetic variants increase the risk for both diseases. After mapping these SNPs to the genome, eight genes were associated with both PD and LBD (Table 2). Notably, *TMEM175* was identified by both PWASs and pleioFDR analysis, indicating its high confidence as a common risk gene for PD and LBD.

**TABLE 2:** Independent genetic loci with a conjFDR < 0.01 in PD and LBD patients identified via pleioFDR analysis.

Locus	SNP	CHR	Position	Gene	ConjFDR PD&LBD	z‐score_PD	z‐score_LBD
1	rs6599389	4	939,113	*TMEM175*	5.74E‐03	4.07	3.88
2	rs1051613	4	951,179	*TMEM175*, *DGKQ*	1.94E‐03	4.90	4.16
3	rs2230288	1	155,206,167	*GBA*, *MTX1P1*	8.51E‐04	4.32	7.04
4	rs1372519	4	90,757,309	*SNCA, SNCA‐AS1*	4.17E‐06	−5.52	−5.48
5	rs11150577	16	29,986,525	*TAOK2, TMEM219*	6.27E‐03	−3.82	−4.04

*Note:* The table provides the results of pleioFDR analysis for five independent genetic loci that are shared between PD and LBD.

Abbreviations: conjFDR, conjunctional false discovery rate; PD, Parkinson’s disease; LBD, Lewy body dementia; SNP, single‐nucleotide polymorphism; CHR, chromosome.

### 3.3 Differential Expression Analysis Confirmed the Downregulation of TMEM175 in Disease States

**FIGURE 3:**
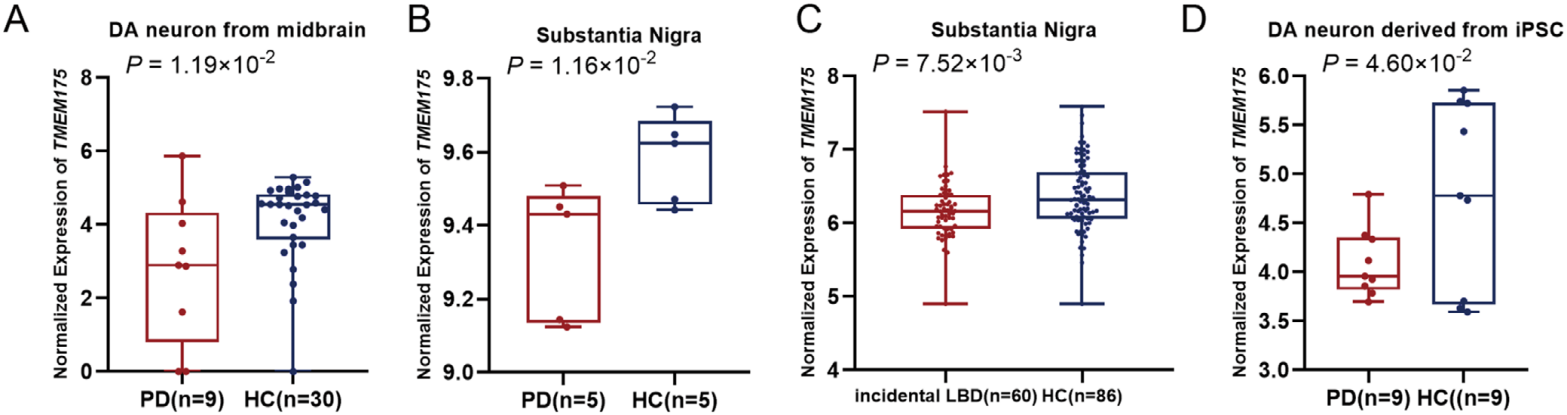
Differential expression analysis of common risk genes between patients and healthy controls validated the downregulation of *TMEM175* in the brain and iPSCs. (A) *TMEM175* was significantly downregulated in DA neurons from the midbrain of PD patients compared with healthy controls. (B) *TMEM175* was dramatically downregulated in the substantia nigra of PD patients compared with healthy controls. (C) *TMEM175* was significantly downregulated in the substantia nigra of incidental LBD patients compared with healthy controls. (D) *TMEM175* was dramatically downregulated in DA neurons derived from iPSCs from PD patients compared with those derived from healthy controls. DA neuron, dopaminergic neuron; HC, healthy control; incidental LBD, incidental Lewy body disease; iPSC, induced pluripotent stem cell; n, number of samples; PD, Parkinson's disease.

To verify the downregulation of *TMEM175* at the transcriptional level in disease states, the expression of RNA in the human brain and iPSCs was examined in patients and healthy controls. First, we investigated the expression of *TMEM175* in human midbrain tissue. In DA neurons isolated from the midbrains of PD patients and healthy controls, *TMEM175* (*p* = 1.19E‐02) was significantly downregulated in the direction of effect, which was consistent with the findings in the PWAS (indicated by negative z scores) (Figure 3A). We subsequently focused on the expression of *TMEM175* in the substantia nigra of patients and healthy controls to further explore the specific brain region in which *TMEM175* is dysregulated. In PD patients, *TMEM175* (*p* = 1.16E‐02) was significantly downregulated in the substantia nigra (Figure 3B). For patients with incidental Lewy body disease, which may represent a preclinical PD or LBD state [39, 40], a similar expression pattern was observed (*p* = 7.52E‐03) (Figure 3C). In addition, in DA neurons differentiated from iPSCs derived from PD patients with *PARK2* mutations, the expression of *TMEM175* was significantly downregulated (*p* = 4.60E‐02) (Figure 3D), whereas no significant difference was detected in neural progenitor cells. Thus, the confirmed downregulation of *TMEM175* at the transcription level suggested that the risk gene *TMEM175* confers disease risk via decreased transcription of *TMEM175*.

## 4 Discussion

Recent studies have identified both shared and disease‐specific genetic loci across various neurodegenerative diseases. For example, a computational analysis of five neurodegenerative disorders—amyotrophic lateral sclerosis (ALS), Alzheimer's disease (AD), PD, multiple sclerosis, and LBD—revealed common genetic factors as well as unique loci associated with each condition [41, 42]. Among these diseases, shared genetic architecture has been identified between PD and LBD [19, 43]; however, the susceptibility genes that are associated with both phenotypes and how they confer disease risk remain largely unknown. In the present study, we elucidated a shared genetic basis between PD and LBD. Specifically, PWASs integrating pQTLs from the human DLPFC with large‐scale GWAS data identified risk proteins for PD and LBD, including 12 proteins for PD and nine proteins for LBD, among which *TMEM175* and *DOC2A* overlapped. Evidence of polygenic overlap between PD and LBD was observed with hierarchical Q–Q plots and five common genetic loci for both diseases were identified utilizing pleioFDR analysis. Among these independent loci, the SNPs rs6599389 [44] and rs2230288 [45, 46] were significantly associated with PD, and rs2230288 [8] was associated with LBD. After mapping to the genome, we obtained eight common risk genes, including four previously reported genes (*TMEM175*, *DGKQ*, *GBA*, and *SNCA*) and four novel susceptibility genes (*MTX1P1*, *SNCA‐AS1*, *TAOK2*, and *TMEM219*). Briefly, *TMEM175*, a lysosomal K^+^ channel; *GBA*, a lysosomal enzyme glucocerebrosidase (GCase) that maintains glycosphingolipid homeostasis; and *SNCA*, circulating α‐synuclein, were previously linked to both PD and LBD [9, 19, 47, 48]. *DGKQ*, a diacylglycerol kinase, was previously implicated in PD [48]. The remaining mapped genes, namely, *MTX1P1*, *SNCA‐AS1*, *TAOK2*, and *TMEM219*, are novel shared risk genes for PD and LBD. Peripheral blood biomarkers are gaining importance in neurodegenerative disease research owing to their accessibility and suitability for large‐scale screening [49] and can potentially be used to identify early PD patients before clinical symptoms appear [50]. Some of the genes we identified, such as *SNCA* [51] and *GBA* [52], have been proposed as promising peripheral markers for PD. Interestingly, the risk protein *TMEM175* was associated with two diseases in both the PWAS and pleioFDR analyses. Finally, we validated the downregulation of *TMEM175* in the substantia nigra and iPSCs using the expression datasets of patients and healthy controls. These results revealed a common polygenic architecture between PD and LBD, which may further our understanding of the relationships between different phenotypes of Lewy body diseases.

Our results strongly supported *TMEM175* as a shared risk gene for PD and LBD. In previous GWASs, *TMEM175* was traditionally considered a PD susceptibility gene and was associated with the age at onset of PD [53, 54, 55]. A recent GWAS revealed that *TMEM175* is an LBD risk gene using multitrait analysis [19]. Moreover, a transcriptome‐wide association study revealed an association between *TMEM175* and LBD [19] that showed the same direction as that reported in the present study. However, whether *TMEM175* confers both PD and LBD risk has not been addressed to date. We provide evidence that *TMEM175* is a common genetic locus in PD and LBD, including SNP, mRNA, and protein levels, and propose that the downregulation of this gene is associated with a greater risk for both diseases. *TMEM175* codes for a potassium channel that regulates lysosomal membrane potential, pH stability, and organelle fusion [56]. *TMEM175* deficiency impairs lysosomal autophagosome clearance and reduces mitochondrial respiration [57]. Association studies and structure prediction have suggested that *TMEM175* may confer PD risk by affecting the activity of glucocerebrosidase [58]. This hypothesis has been confirmed in rat primary neurons, in which *TMEM175* deficiency promoted α‐synuclein deposition and reduced the enzymatic activity of the lysosomal gene *GBA* [57]. However, the results of animal experiments are still controversial. *TMEM175*‐knockout mice presented a significant reduction in the number of DAergic neurons in the substantia nigra compacta and impaired motor function [59]. In another study, knockdown of *TMEM175* mitigated motor impairment and DA neuron loss in a 1‐methyl‐4‐phenyl‐1,2,3,6‐tetrahydropyridine (MPTP)‐induced mouse model [60]. More studies are needed to explore the impact of the dysregulation of *TMEM175* expression in different animal disease models in the future. In terms of drug development, *TMEM175* is a potential drug target, as the cryo‐EM structure of human *TMEM175* has been revealed recently [61]. Novel therapies based on the interactions between TMEM175 and other proteins may reverse the impairment of lysosomal function caused by pathogenic variants [59, 60].

In addition to *TMEM175*, the common risk loci *SNCA*, *GBA*, and *DGKQ* discovered by pleioFDR analysis are classical PD risk genes [62], whereas *SNCA* and *GBA* are associated with LBD in GWASs [9]. Beyond our findings in PD and LBD, *TMEM175*, *SNCA*, and *GBA* were recently identified as risk genes for rapid eye movement sleep behavior disorder (RBD) [63], an early clinical manifestation of α‐synuclein aggregation disorders associated with Lewy bodies, PD, and multiple system atrophy [64]. The biological functions of these genes are intimately linked with α‐synuclein aggregation pathways that involve lysosomal and mitochondrial dysfunction [65, 66, 67], representing common downstream effects of diverse upstream pathologies observed in neurodegenerative diseases. For example, similar impairments have been reported in patients with ad [ 68], ALS [69], and frontotemporal dementia [70]. This convergence suggests that, despite their distinct etiologies, many neurodegenerative disorders may ultimately activate shared pathways, leading to neuron damage.

We also identified novel common risk genes, including *MTX1P1*, *TAOK2*, and *TMEM219*. *MTX1P1* encodes the pseudogene for metaxin, separating GBA from its pseudogene [71]. Owing to a lack of the first intron and promoter region, the expression of this gene cannot be detected in most tissues [71]. Whether *MTX1P1* affects the function of GBA and how *MTX1P1* confers disease risk need to be further explored. *TAOK2* is a serine/threonine kinase associated with autism spectrum disorder [72]. Experimental evidence has revealed the vital role of *TAOK2* in the process of cortical development, including neuron migration and spine maturation [72, 73, 74]. *TMEM219* is a novel receptor that mediates the antitumor effects of insulin‐like growth factor binding protein‐3 [75], but its function in the brain remains unclear. The relationship between *TAOK2/TMEM219* and PD/LBD requires replication by other independent association studies. In addition, *DOC2A*, which was identified in both the PD and LBD PWASs, is a candidate common risk protein. This gene encodes a high‐affinity calcium‐binding protein that affects synaptic transmission by regulating asynchronous neurotransmitter release [76].

Our study has several strengths. First, the integration of diverse data from genome‐wide SNP and proteome analyses enabled us to identify risk proteins, incorporating regulation at the transcription and translation levels. Second, protein weights from the DLPFC could directly reflect dysregulation in situ in disease states and reveal risk factors. Third, the application of differential gene expression analysis using tissues derived from different sources not only validated our results at the transcriptional level but also provided evidence that dysregulation of risk genes already exists in the early stages of the diseases.

Our study also has several limitations. First, ancestry distribution and sample size may affect the statistical power of the PWASs. The present study used only GWAS datasets of PD and LBD patients of European descent, leading to limitations in the conclusions and necessitating future examination of large cohorts with different ancestral backgrounds. Second, pleioFDR is restricted to bivariate analysis, so the effects of other nongenetic factors on genetic overlap cannot be determined. Third, owing to data availability, the dysregulation of risk genes in LBD patients could not be verified in multiple tissue types. Finally, whether the shared genetic components are causal variants and whether they act through the same underlying biological mechanism requires further investigation.

In conclusion, we demonstrated a shared polygenic structure between PD and LBD. These findings have implications for understanding the genetic risk and pathogenesis of complex polygenic neurodegenerative diseases and may provide insights for future research on common drug targets for both diseases.

## Author Contributions

All authors made contributions in our research. C.Z. provided the conception and funding acquisition for the work, and also reviewed and edited the manuscript. T.J. conducted formal analysis and original draft preparation. C.Z., F.Y., F.Q., Y.H., and F.H. participated in formal analysis and visualization. All authors have read and agreed to the published version of the manuscript.

## Conflicts of Interest

The authors declare no conflicts of interest.

## Supporting information


Data S1.

**Figure S1.** Conditional quantile–quantile (Q‐Q) plots indicated cross‐disease genetic enrichment between PD and LBD.

## Data Availability

The data that support the findings of this study are available in AD Knowledge Portal – backend at https://doi.org/10.7303/syn23627957. These data were derived from the following resources available in the public domain: – syn23627957, https://doi.org/10.7303/syn23627957
