# Supplementary material for: Identification of Common Brain Protein and Genetic Loci Between Parkinson's Disease and Lewy Body Dementia
Source: CNS Neurosci Ther. 2025 Apr 9;31(4):e70370. doi: 10.1111/cns.70370 (PMC11979625; doi:10.1111/cns.70370)
Supplement: Supplementary file 1 — Data S1. [file CNS-31-e70370-s002.doc]

**Identification of common brain protein and genetic loci between Parkinson's disease and Lewy body dementia**

**Supplemental Materials**

**Materials and Methods**

***Visualization of cross-disease genetic effect enrichment***

We constructed conditional quantile-quantile (Q-Q) plots to assess cross-disease enrichment of polygenic effect. Conditional Q-Q plots compared associations with one primary trait in the SNP strata of significant association with the conditional trait. In the absence of association between PD and LBD, the Q-Q plot follows a straight dashed line (the expected line). When the systematic association exists, the Q-Q plot deviates leftward continuously from the expected line. Stronger enrichment together with increased evidence of association with the conditional trait indicated a shared risk locus between the two traits.

**
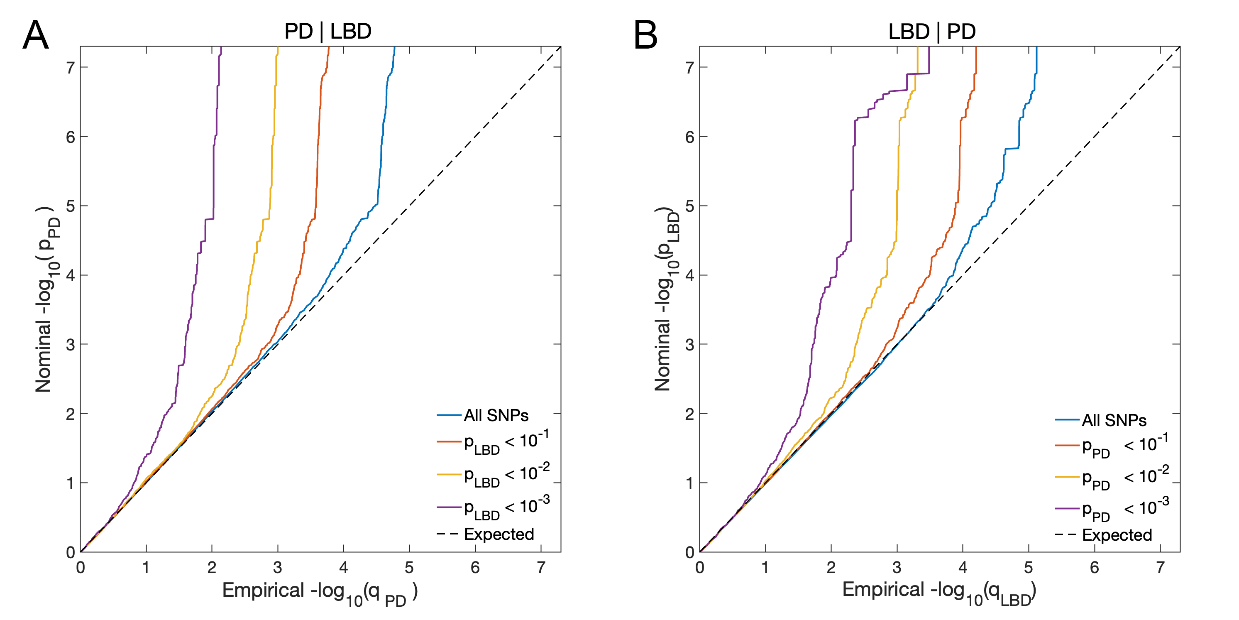
**

**Supplementary figure 1.** Conditional quantile–quantile (Q-Q) plots indicated cross-disease genetic enrichment between PD and LBD.

Q-Q plots showed nominal versus empirical -log10 p-values in the primary trait (disease phenotype) below the standard GWAS p-value threshold of 5×10^-8^ as a function of significance of association with the conditional trait at the level of p<0.1, p<0.01, and p<0.001. The dashed lines indicate the null hypothesis. (A) The primary trait was PD, while the conditional trait was LBD. (B) The primary trait was LBD, and the conditional trait was PD. PD, Parkinson’s disease; LBD, Lewy body dementia; SNP, single nucleotide polymorphism; GWAS, genome-wide association study.

**Results**

**Supplementary Table 1.** A total of 34 SNPs at 5 loci were identified with significant effects (conjFDR < 0.01) in PD and LBD.

| Locus | chr | position | *p*_PD | FDR_PD | conjFDR_PD&LBD |
| --- | --- | --- | --- | --- | --- |
| 1 | 1 | 155206167 | 1.53E-05 | 5.44E-02 | 8.51E-04 |
| 2 | 4 | 939113 | 4.71E-05 | 1.37E-01 | 5.74E-03 |
| 3 | 4 | 941290 | 6.77E-05 | 1.80E-01 | 3.33E-03 |
| 3 | 4 | 951040 | 9.59E-05 | 2.26E-01 | 4.57E-03 |
| 3 | 4 | 951179 | 9.39E-07 | 3.84E-03 | 1.94E-03 |
| 3 | 4 | 965779 | 3.20E-05 | 1.01E-01 | 7.59E-03 |
| 4 | 4 | 90682504 | 1.87E-09 | 1.00E-05 | 3.84E-04 |
| 4 | 4 | 90688120 | 4.90E-09 | 2.54E-05 | 2.99E-05 |
| 4 | 4 | 90691537 | 4.95E-09 | 2.57E-05 | 1.91E-05 |
| 4 | 4 | 90705364 | 1.79E-07 | 7.78E-04 | 4.29E-05 |
| 4 | 4 | 90718719 | 5.32E-09 | 2.76E-05 | 3.87E-05 |
| 4 | 4 | 90719192 | 5.31E-09 | 2.75E-05 | 3.87E-05 |
| 4 | 4 | 90729602 | 5.33E-09 | 2.77E-05 | 3.87E-05 |
| 4 | 4 | 90729747 | 5.32E-09 | 2.76E-05 | 3.00E-05 |
| 4 | 4 | 90736006 | 6.37E-08 | 3.04E-04 | 7.26E-03 |
| 4 | 4 | 90736113 | 5.86E-09 | 3.03E-05 | 3.94E-05 |
| 4 | 4 | 90736727 | 6.73E-08 | 3.22E-04 | 9.09E-03 |
| 4 | 4 | 90739255 | 3.87E-07 | 1.62E-03 | 9.41E-03 |
| 4 | 4 | 90740878 | 1.05E-06 | 4.25E-03 | 8.18E-03 |
| 4 | 4 | 90746646 | 8.47E-09 | 4.31E-05 | 1.77E-05 |
| 4 | 4 | 90747751 | 8.43E-09 | 4.29E-05 | 2.25E-05 |
| 4 | 4 | 90748374 | 8.41E-09 | 4.27E-05 | 2.25E-05 |
| 4 | 4 | 90748488 | 3.73E-08 | 1.83E-04 | 1.71E-05 |
| 4 | 4 | 90750326 | 8.41E-09 | 4.27E-05 | 2.46E-05 |
| 4 | 4 | 90754313 | 7.86E-09 | 3.99E-05 | 1.05E-05 |
| 4 | 4 | 90757309 | 3.35E-08 | 1.64E-04 | 4.17E-06 |
| 4 | 4 | 90784528 | 1.30E-06 | 5.27E-03 | 9.55E-05 |
| 5 | 16 | 29967434 | 1.60E-04 | 2.99E-01 | 7.25E-03 |
| 5 | 16 | 29968015 | 1.69E-04 | 3.08E-01 | 7.65E-03 |
| 5 | 16 | 29974167 | 1.70E-04 | 3.09E-01 | 7.68E-03 |
| 5 | 16 | 29986525 | 1.36E-04 | 2.75E-01 | 6.27E-03 |
| 5 | 16 | 29988941 | 1.67E-04 | 3.06E-01 | 7.57E-03 |
| 5 | 16 | 29995218 | 1.80E-04 | 3.18E-01 | 8.11E-03 |
| 5 | 16 | 29995880 | 1.80E-04 | 3.18E-01 | 8.10E-03 |

The table showed 34 SNPs and their corresponding conjFDR in the Manhattan plot (Figure 2). PD, Parkinson’s disease; LBD, Lewy body dementia; chr, chromosome.
