# Supplementary figures and images for: Identification of Common Brain Protein and Genetic Loci Between Parkinson's Disease and Lewy Body Dementia
Source: CNS Neurosci Ther. 2025 Apr 9;31(4):e70370. doi: 10.1111/cns.70370 (PMC11979625; doi:10.1111/cns.70370)

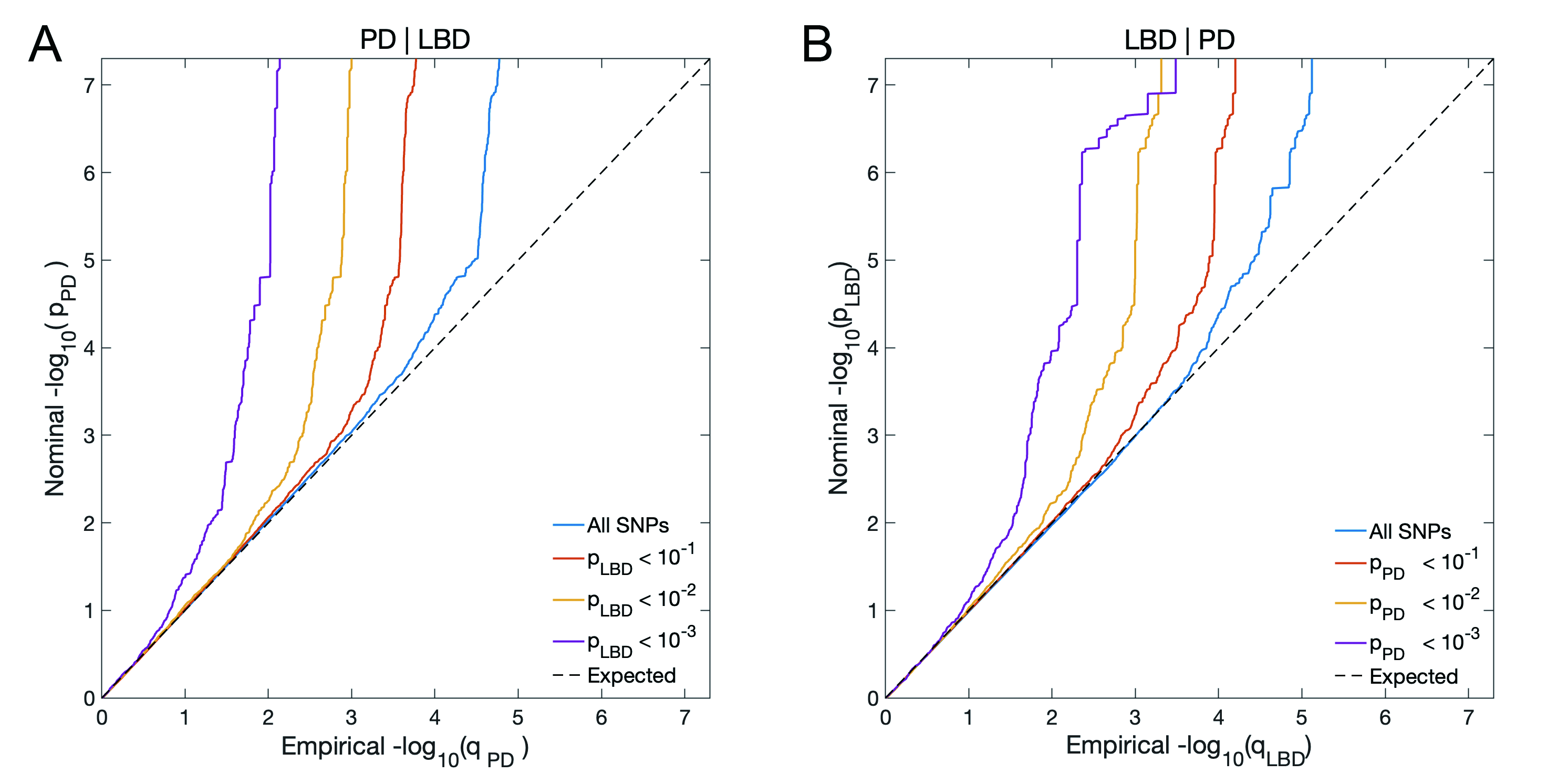

Supplement: Supplementary file 2 — Figure S1. Conditional quantile–quantile (Q‐Q) plots indicated cross‐disease genetic enrichment between PD and LBD. [file CNS-31-e70370-s001.tif]
